# The Predictive Role of ADRA2A rs1800544 and HTR3B rs3758987 Polymorphisms in Motion Sickness Susceptibility

**DOI:** 10.3390/ijerph182413163

**Published:** 2021-12-14

**Authors:** Xinchen Zhang, Yeqing Sun

**Affiliations:** Institute of Environmental Systems Biology, Dalian Maritime University, Dalian 116023, China; bfdplmxs@yeah.net

**Keywords:** motion sickness susceptibility, vomiting, ADRA2A rs1800544, HTR3B rs3758987, sailing environment

## Abstract

Motion sickness is a common central nervous system response, the primary sign of which is vomiting. Its susceptibility varies between individuals. To find predictive factors, we investigated the association of ADRA2A rs1800544 and HTR3B rs3758987 with motion sickness susceptibility and examined their mRNA changes during actual voyages. A total of 315 healthy college students were enrolled for SNP genotyping by the PCR-RFLP method. Blood samples were collected from another 42 subjects during two separate voyages to detect their mRNA expression changes at three time points. The frequency of the rs1800544 GG genotype in the susceptibility group was significantly higher (52.26%), and allele G increased the risk of motion sickness (OR = 1.585, 95% CI = 1.136–2.208). In the logistic regression model, the rs3758987 CC+TC genotype and rs1800544 GG genotype increased the risk of motion sickness-induced vomiting (OR = 2.105, 95% CI = 1.112–3.984; OR = 1.992, 95% CI = 1.114–3.571). The ADRA2A mRNA baseline was lower in the GG carriers and the HTR3B mRNA baseline was lower in the TC/CC carriers before sailing, then increased significantly within 24 h and then decreased after a long-term voyage. People carrying the rs1800544 GG genotype seem more susceptible to motion sickness. In combination with the incidence of vomiting during the actual-voyage experiments, our results indicate the involvement of rs1800544 and rs3758987 in motion sickness-induced vomiting.

## 1. Introduction

Motion sickness (MS) is a central nervous system (CNS) response to an unfamiliar motion stimulus, which can be provoked by a variety of transport environments. It is denominated by the form of provocative environments, such as seasickness, carsickness, airsickness, space sickness, simulator sickness, and virtual reality sickness, among others, and the most serious form is seasickness [1]. When the brain receives discrepant information from different sensors (eyes or vestibular apparatus), the conflicting sensory information will cause a mismatch between the afferent motion perception information and the experienced motion conditions stored in the brain, which initiates motion sickness within minutes [2,3]. The passive motion signals transmit to the vestibular nuclei, and then through the cerebellum to diverse areas of the central and peripheral nervous systems, resulting in different symptoms [4,5,6]. The initial symptom of MS is usually “stomach awareness”, and then followed by a series of signs such as nausea and vomiting [7,8]. The development of MS varies with the stimulus intensity and individual susceptibility, which is thought to be a result of genetic–environment interaction [9].

MS is very common among the general population, especially among practitioners engaged in occupations related to aviation, aerospace, and maritime affairs. Physiological dysfunction and discomfort caused by MS may lead to cognitive decline and impaired judgment, which increase the accident risks for practitioners who are exposed to aviation and marine environments [10]. Therefore, a study for finding the predictors of motion sickness susceptibility (MSS) is of great value for the health of these practitioners. Individual susceptibility to motion sickness varies greatly and is affected by many factors. Previous studies revealed that it was influenced by age and gender, such that younger people and women were more prone to MS [11,12]. Our previous research had found that subjects who were born and living in inland provinces of China were more susceptible to motion sickness [13]. Most studies used the motion sickness susceptibility questionnaires (MSSQ) to evaluate the individual susceptibility [14]. The average predictive validity of the MSSQ was around r = 0.45, which is not as high as expected. The reasons for this probably include subjective bias, understanding differences, and the very different histories of motion exposure for the individuals, as well as the multifactorial nature of motion sickness susceptibility itself. Therefore, it may be more reliable to use genetic polymorphisms to predict the susceptibility of motion sickness.

MS is a conserved cross-species phenotype with a high heritability in humans [15]. A classic concordance twin study showed that the heritability of MSS was 60–70% [16]. A study that enrolled 80,494 participants found 35 single nucleotide polymorphisms (SNPs) in genes involved in multiple functions that were associated with self-reported carsickness susceptibility [17]. A study suggested that MSS was related to individual differences in the autonomic responses caused by genetic polymorphisms [18]. These findings provide insights about how MSS may be influenced by some key SNPs in genes involved in the transduction and reception of the motion signal pathways. However, because of the different simulated environments and complex sample sources of previous researches, the key gene variants of motion sickness are still inconclusive at present.

MSS is associated with increased sympathetic and decreased parasympathetic activities [19]. The α_2A_-adrenergic receptors (ADRA2A) regulate the norepinephrine release and cause sympathetic activation [20]. It is suggested that some polymorphisms of ADRA2A may lead to MSS differences. Liu et al. found that the GG genotype of the ADRA2A-1296 site might be relevant to the hyper-motion sickness susceptibility of Asians [21], and Finley et al. reported that the Dra I RFLP in ADRA2A was associated with the individual differences in the autonomic response to stress provoked by motion [18]. The ADRA2A rs1800544 is reported to be associated with depression [22], attention-deficit/hyperactivity disorder [23], and an escape from dexamethasone and elevated glucose levels [24], which are all related to the catecholamine pathway and the sympathetic nervous system imbalance. The α-2a adrenergic receptor mediates the central and peripheral autonomic responses to stress, such as sweating and nausea. These responses may be “downstream” from the peripheral vestibular inputs [18]. The rs1800544 is in the promoter region of ADRA2A [25]. It is important to understand the potential function of the polymorphism in motion sickness. Of course, because of the complex mechanism of MS, it still needs a lot of research in the future.

Vomiting is the typical symptom of MS [26]. Generally, stimulus signals are transmitted from the intestines to the brain through nerves, activating the vomiting center and causing the individual to vomit [27,28]. The enteric nervous system (ENS) is now considered a third component of the autonomic nervous system. It delicately regulates the function of several target organs, including the gastrointestinal tract, resulting in a series of symptoms [29]. The 5-hydroxytryptamine type 3B receptor (HTR3B) plays an important role in this progress [30]. The 5-HT3 receptor mediates vomiting, which occurs via the activation of Ca2+/CaMKII-dependent ERK1/2 signaling [27]. Genetic polymorphisms of the HTR3B may affect the expression or function of the HTR3 complex, and the signaling of serotonin. Some HTR3B SNPs were reported to be associated with susceptibility to postoperative and chemotherapy-induced nausea and vomiting (PONV and CINV) [31,32]. The −100_−102delAAG deletion variant and Tyr/Tyr genotype of Tyr129Ser in HTR3B were associated with a higher risk of CINV and paroxetine-induced nausea [33,34,35]. A previous study found that the HTR3B rs3758987 might serve as a predictor of PONV in Chinese Han patients undergoing gynecological laparoscopic surgery [36]. Since vomiting shares the ‘final common pathway’, genetic variants that affect PONV and CINV may also contribute to MS-induced vomiting.

In summary, the SNPs of ADRA2A (transduction) and HTR3B (reception) genes may play a role in predicting an individual’s motion sickness susceptibility, and clarifying their predictive functions is of great significance. These receptors were mostly distributed in the CNS and ENS, but the use of brain or gastrointestinal biopsies of living patients is unrealistic for biochemical investigation, therefore the peripheral blood may be a convenient and accessible alternative. The previous studies showed that peripheral gene expression may be a useful potential surrogate for gene expression in the brain [37,38]. The genes expressed in the brain are also expressed in peripheral blood cells, and some show co-expression or similar expression in the same individuals, supporting their use as a surrogate tissue for gene expression in the brain [39]. Blood cells, especially lymphocytes, may act as convenient indicators for the expression of catecholamine and serotonin receptor genes [40]. Therefore, we collected the blood samples of subjects for detecting their gene expression.

To explore the association between genetic background and motion sickness susceptibility, we designed a two-part experiment. In part one, we randomly extracted 350 subjects from the 1051 questionnaire participants of our previous study [13] to analyze the association between the ADRA2A rs1800544 and HTR3B rs3758987 polymorphisms and motion sickness susceptibility. In part two, we recruited 42 subjects to participate in two separate actual-voyage experiments to examine the ADRA2A and HTR3B mRNA dynamic changes in the subjects with each type of genotype for these SNPs under different sea states. This study aims to investigate the association between ADRA2A rs1800544 and HTR3B rs3758987 and motion sickness, obtain predictive genotypes, explore the dynamic expression changes of these two genes in different genotypes in actual voyages, and to find possible biomarkers for the prediction of seasickness and to explore its possible habituation or protective factors.

## 2. Materials and Methods

### 2.1. Study Population and Design

All the subjects enrolled in this study were Chinese Han students from Dalian Maritime University (Liaoning, China). In part one, 350 subjects (269 males, 81 females; mean age: 18.32 ± 0.65 years) were randomly selected from 1051 subjects of our previous study [13]. They were asked to complete the MSSQ to initially evaluate their susceptibility to motion sickness. The MSSQ collects information about how often the subjects felt ‘sick or nauseated’ and ‘vomited’ during childhood (before 12 years) and adulthood (in the last 10 years), respectively, when exposed to nine forms of transportation and entertainment [14]. In this study, we defined motion sickness susceptibility by their MSSQ score, where <22.68 was designated as the non-susceptibility group and ≥22.68 was the susceptibility group, based on our previous work [13]. We extracted the ‘vomited’ part for further analysis and distinguished the subjects as vomited and non-vomited groups in both childhood and adulthood parts, respectively. We then collected their blood samples for genotyping. Finally, 315 samples were enrolled because of hemolysis and other reasons.

In part two, another 42 male students (mean age: 21.12 ± 1.10 years) without navigational experience took part in the sailing experiment. This was performed as part of the voyage of an annual training event for interns on the ship *YuKun*. Before they sailed, the subjects were asked to complete the MSSQ, and their blood samples were collected for the first time. A total of 27 subjects took part in the first voyage, which started at 15:00 on 21 November 2016 from the Port of Dalian, and ended on 3 December 2016, lasting 12 days. During this voyage, the weather was generally clear, and the sea state held on degree 2~3 (wave height: 0.5~1.0 m) according to the ocean weather forecast reported by the Meteorological Bureau of Liaoning Province. We collected their Graybiel motion sickness questionnaire (GMSQ) data on the ship at 15:00 on days two and ten and collected their blood samples at the same time. GMSQ was used to assess the seasickness symptoms during the voyage. The higher the score on the GMSQ, the more serious the seasickness is [41]. The other 15 subjects took part in the second voyage, which started at 9:00 on 18 December 2016 and ended on 25 December 2016, lasting seven days. The weather was calm during the first three days (degree 2~3) but became windy on day 4 (degree 5, wave height: 2.0~2.5 m). Therefore, we collected the GMSQ data and their blood samples at 15:00 on days two and five as an additional stimuli group. All the subjects were asked not to take any medication during the whole voyage. Before the experiment started, we obtained written informed consent from each subject. This study was conducted in accordance with the Declaration of Helsinki, and the protocol was approved by the Ethics Committee of Dalian Maritime University, Liaoning, China. (Project identification code: DLMU-IESB-2016-001).

### 2.2. DNA Sampling and Genotyping

All the blood samples were collected in ethylenediaminetetraacetic acid-Na-containing tubes. The blood cells were then centrifuged out at 1500 rpm for 5 min and stored at −80 °C. The genomic DNA was extracted using a FastPure Blood DNA Isolation Mini Kit (Vazyme, Nanjing, China) according to the manufacturer’s protocol. The genotype of ADRA2A (rs1800544) and HTR3B (rs3758987) were detected by the polymerase chain reaction-restriction fragment length polymorphism method (PCR-RFLP). Following the endonuclease digestion, the PCR products were resolved through 2.5% agarose gel electrophoresis and visualized by ethidium bromide staining.

The ADRA2A fragment was 229bp, and its primer sequences were 5′-GGTTACTTCCCTCGATTTGGG-3′ and 5′-GGGACGAGCCCTTTGGAGA-3′. The detailed cycling conditions were as follows: an initial denaturation step at 94 °C for 1 min; then 94 °C for 15 s, 55 °C for 30 s, and 72 °C for 30 s for 30 cycles; then extended at 72 °C for 2 min; and finally stored at 4 °C. The genotypes were CC (171+58 bp), GC (171+116+55+58 bp), and GG (116+55+58 bp) after the Msp I digestion. The primer sequences for HTR3B were 5′-TTCAAGAGCCCAAGAACCACT-3′ and 5′-AATGCCGCTCAATTTCTCC-3′, with a length of 449 bp. The detailed cycling conditions were as follows: an initial denaturation step at 95 °C for 1 min; then 95 °C for 30 s, 56 °C for 30 s, and 72 °C for 30 s for 35 cycles; then extended at 72 °C for 4 min; and finally stored at 4 °C. The genotypes were CC (449 bp), TC (449+331+118 bp), and TT (331+118 bp) after the Sca I digestion (Figure S1). We randomly chose 10% of the samples for re-genotyping to examine the accuracy, and the coincidence rate was close to 99%.

### 2.3. Real-Time Quantitative Polymerase Chain Reaction (qRT-PCR)

Total RNA was homogenized and purified by a TRIzol reagent (Invitrogen, Carlsbad, CA, USA). The retrotranscription reaction was performed by the PrimeScript™ RT reagent Kit with a gDNA Eraser (Perfect Real Time) (TaKaRa). The qRT-PCR reaction was performed in a LightCycler^®^ 480 PCR machine (Roche, Basel, Switzerland), and the detailed cycling conditions were as follows: an initial denaturation step at 95 °C for 5 s; then 95 °C for 15 s and 60 °C for 60 s for 40 cycles. All values were normalized to the housekeeping gene h-GAPDH, and the 2^−ΔΔCT^ method was used to evaluate the relative expression of the mRNAs. The primer pairs for ADRA2A were 5′-AGAAGTGGTACGTCATCTCGT-3′ and 5′-CGCTTGGCGATCTGGTAGA-3′, and for HTR3B they were 5′-TTACAACTGGACCAAGGCCACCA-3′ and 5′-TTGATTCTCTGCATCCACATCC-3′. All the reactions were run in triplicate, and all the experiments were repeated three times. The coefficient of variation (CV), indicating the ratio of SD and mean in percent, was calculated to evaluate the precision and repeatability of the experiments. Except for a few outliers with higher CVs, most of the experiments showed a CV < 20%. In this study, the mean intra- and inter-assay coefficients of variation were 6.31% and 12.63% for the ADRA2A mRNA expression, and were 5.75% and 11.50% for the HTR3B mRNA expression.

### 2.4. Statistical Analysis

The statistical analysis was completed using the IBM SPSS Statistics 22.0 program. The Levene test and Shapiro–Wilk test were used to assess the normality and homogeneity of the variance for the variables. If the variable was skewed, before further analysis, it was adjusted to a normal distribution through a square root transformation. A Hardy–Weinberg equilibrium test was used to assess the hereditary deviations, and if *p* > 0.05, the frequency conforms with the HW equilibrium law. The categorical variables were expressed by frequency counts. The Chi-square test was used to analyze the relationship between MSS, the symptoms, and the rs1800544 and rs3758987 polymorphisms. A logistic regression was used to assess the risks of motion sickness susceptibility in the different genotypes. An independent sample t-test and paired t-test were used for the analysis of the dynamic changes of ADRA2A and HTR3B mRNAs. There was a statistically significant difference when *p <* 0.05. The significance for the two SNPs located in the different genes was 0.025. The G∗Power 3.1 software (Heinrich-Heine-Universität, Germany) was used for a *post hoc* power analysis of the most statistically significant results with an α error probability set at 0.05 [42].

## 3. Results

### 3.1. Demographic Characteristic of Population and Hardy-Weinberg Genetic Equilibrium Test

In part one, a total of 315 samples were enrolled for the SNP genotyping analysis because of the hemolysis, among other reasons. The demographic characteristics of the subjects and the percentages of variables were listed in Table 1. Gender and MSSQ scores were not statistically different between age groups. The mean MSSQ score of the subjects was 29.25 ± 32.62, with a positively skewed distribution. We defined the motion sickness susceptibility of the subjects by their MSSQ scores, where <22.68 was designated as the non-susceptibility group, and ≥22.68 was the susceptibility group, according to our previous work [13]. Based on this criterion, we obtained 155 subjects as the susceptibility group, and 160 subjects as the non-susceptibility group.

Among the 315 subjects, the frequency of the GG, GC, and CC genotype was 44.13% (139), 42.54% (134), and 13.33% (42) in ADRA2A rs1800544, where allele C was 34.6% and G was 65.4%. Its MAF was 0.35 [(134 + 42 × 2)/315 × 2]. With regards to HTR3B rs3758987, the frequency of the TT, TC, and CC genotype was 62.54% (197), 33.65% (106), and 3.81% (12), where allele C was 20.6% and T was 79.4%. The MAF of rs3758987 was 0.21 [(106 + 12 × 2)/315 × 2]. The distribution of the actual value and expected value had no significant difference in both the rs1800544 and rs3758987 genotypes (χ^2^ = 0.549, *p =* 0.760; χ^2^ = 0.084, *p =* 0.959), which indicates that their distributions were consistent with Hardy–Weinberg’s law of genetic equilibrium, and were representative of the general population.

### 3.2. The ADRA2A rs1800544 and HTR3B rs3758987 Polymorphism and Risk of Motion Sickness Susceptibility

There were significant differences in the distribution of the ADRA2A rs1800544 genotypes between the susceptibility and non-susceptibility groups, and the distribution frequency of the GG genotype in the susceptibility group increased significantly. In the analysis of the dominant model, the distribution of GG in the susceptibility group was significantly higher than in the non-susceptibility group. The relative-risk analysis showed that the CC+GC genotype reduced the risk of MS (OR = 0.519, 95%CI = 0.331–0.815). The distribution frequency of the G allele in the susceptibility group was 70.6%, which was significantly higher than in the non-susceptibility group, and the G carriers had a higher risk of MS (OR = 1.585, 95%CI = 1.136–2.208; Table 2). There was no significant association between the MSS and HTR3B rs3758987.

### 3.3. The ADRA2A rs1800544 and HTR3B rs3758987 Polymorphism and Risk of Motion Sickness-Induced Vomiting

We distinguished the subjects as vomited group/non-vomited group in childhood and adulthood, respectively, according to their answers on the ‘vomited’ part in the MSSQ. In the current study, we obtained 181 subjects as the vomited group (134 as the non-vomited group) in childhood and 125 subjects as the vomited group (190 as the non-vomited group) in adulthood. Table 3 shows the data of the childhood part. The frequency of the HTR3B rs3758987 TC genotype was significantly higher in the vomited group than in the non-vomited group. The distribution frequency of the genotypes in the adulthood part had the same trend, but was weaker than in the childhood part (*p =* 0.064). In the dominant model analysis, the CC+TC genotype increased the risk of MS-induced vomiting with reference to the TT genotype (OR = 1.679, 95%CI = 1.049–2.690). For ADRA2A rs1800544, the frequency of the GG genotype was significantly higher in the vomited group. The CC+GC genotype reduced the risk of MS-induced vomiting in the dominant model analysis (OR = 0.615, 95%CI = 0.390–0.970). The distribution frequency of the G allele in the vomited group was significantly higher, and the G carriers had a higher risk of vomiting (OR = 1.504, 95%CI = 1.079–2.092). The result suggested that these two variants might have some predictive functions for MS-induced vomiting.

### 3.4. Logistic Regression Analysis of Risk of Motion Sickness Susceptibility and MS-Induced Vomiting

**Figure 1:**
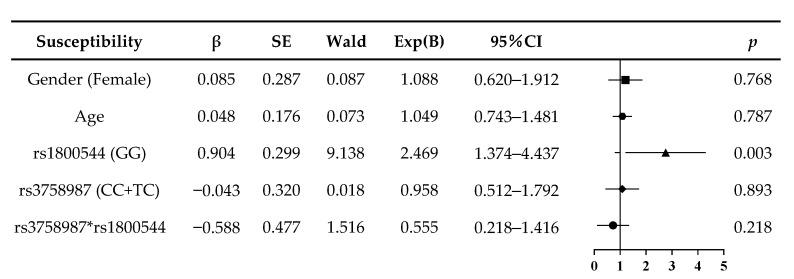
Logistic regression analysis of risk of motion sickness susceptibility in subjects.

**Figure 2:**
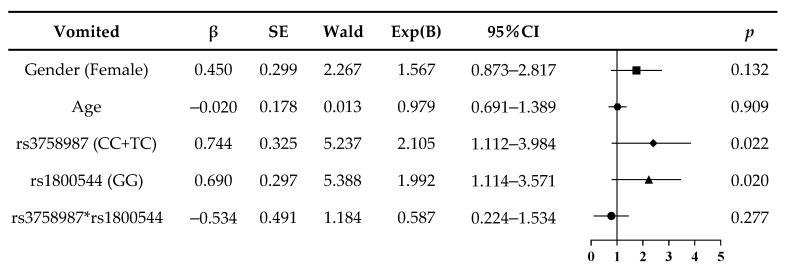
Logistic regression analysis of risk of motion sickness induced-vomiting in subjects.

The combined genotype analysis was performed by a logistic regression. Figure 1 shows that the rs1800544 GG genotype was an independent risk factor of motion sickness susceptibility (Exp(B) = 2.469, *p =* 0.003; Figure 1). The risk factors of MS-induced vomiting were the rs3758987 CC+TC and rs1800544 GG genotypes (Exp(B) = 2.105, *p =* 0.022; Exp(B) = 1.992, *p =* 0.020; Figure 2). Since our subjects were in the same age group, and their age difference was no more than one year, the age factor in the logistic regression model was not significantly different. For the gender factor, there was a trend amongst the female subjects that suggests they are more prone to motion sickness and vomiting, but the difference was not significant enough, which may be because of the relatively small sample size of this study.

### 3.5. Demographic Characteristic and Genotype Frequency of Subjects in Actual Voyages

In part two, we recruited another 42 subjects for the two separate actual-voyage experiments, 27 for the first voyage and 15 for the second voyage. Among them, 11 subjects (26.2%) were carrying GG, 30 (71.4%) were carrying GC, and 1 (2.4%) was carrying the CC genotype in ADRA2A rs1800544. For rs3758987, there were 26 subjects (61.9%) carrying TT, 14 (33.3%) for TC, and 2 (4.8%) for the CC genotype. The constituent ratios of the subjects had no significant difference between parts one and two, and the frequencies of the SNPs conformed with the Hardy–Weinberg equilibrium law. Given that all the subjects had no sailing experience, and the sea state degrees were not significantly different during the first 24 h between the two voyages, the data obtained on day two were pooled for further analysis. Nearly 93% of the subjects had some level of seasickness symptoms within 24 h, and 16 subjects vomited and were defined as the vomited group. The frequency of the rs3758987 CC+TC genotype was significantly higher in the vomited group (62.5%) than in the non-vomited group (23.1%; *p =* 0.010). Furthermore, the distribution frequency of the rs1800544 GG genotype was higher in the vomited group (31.3%) than in the non-vomited group (23.1%), but not significantly (*p =* 0.56).

### 3.6. Basic Expression ADRA2A and HTR3B mRNA in Different Genotype Carriers

**Figure 3:**
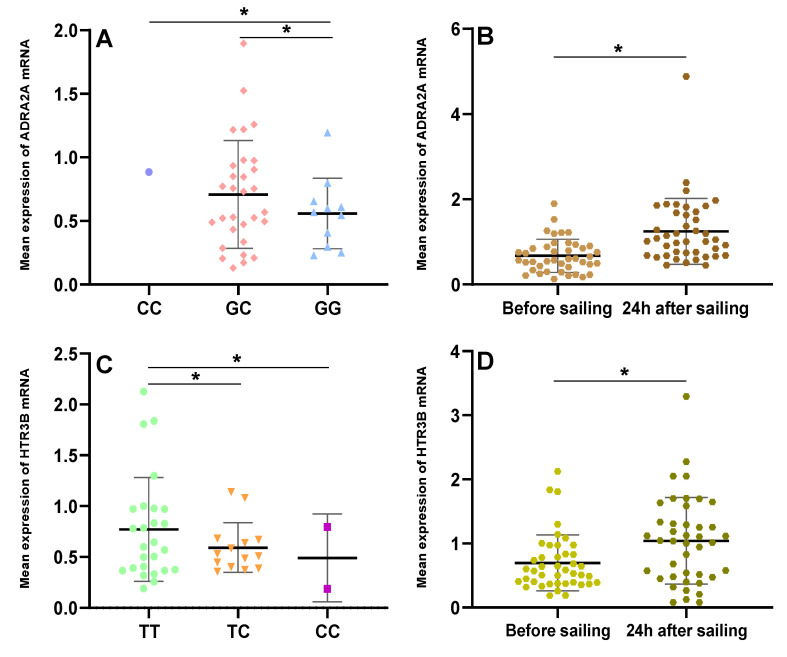
Basic level and changes within 24 h of ADRA2A and HTR3B mRNA in subjects in different genotypes. (**A**) Basic ADRA2A mRNA level in different rs1800544 genotypes; (**B**) Changes of ADRA2A mRNA level during 24 h of voyage in all subjects; (**C**) Basic HTR3B mRNA level in different rs3758987 genotypes; (**D**) Changes of HTR3B mRNA level during 24 h of voyage in all subjects. * *p <* 0.05; error bar: Standard Deviation (SD).

We investigated the influence of exposure to sailing at sea on the ADRA2A and HTR3B mRNA level changes in the subjects with each type of genotype for the examined SNPs (rs1800544 and rs3758987). We collected the blood samples of all 42 subjects seven days before their sailing and found that the basic ADRA2A mRNA level was significantly lower in the rs1800544 GG carriers than in the GC and CC carriers (*p <* 0.05; Figure 3A). Furthermore, for the HTR3B mRNA, its basic level of rs3758987 TT carriers was significantly higher than that of the TC and CC carriers (*p <* 0.05; Figure 3C). Within the first 24 h of the two voyages, all 42 subjects were exposed to the same stimuli by the voyage environment. Compared to the basic level, the ADRA2A and HTR3B mRNAs were both significantly increased within 24 h of the voyage (*p =* 0.000; *p =* 0.001; Figure 3B,D). The mRNA expressions (Mean ± SD) were shown in Table S1.

### 3.7. Dynamic Changes of ADRA2A and HTR3B mRNAs of Subjects during Two Voyages under Different Sea State Degrees

**Figure 4:**
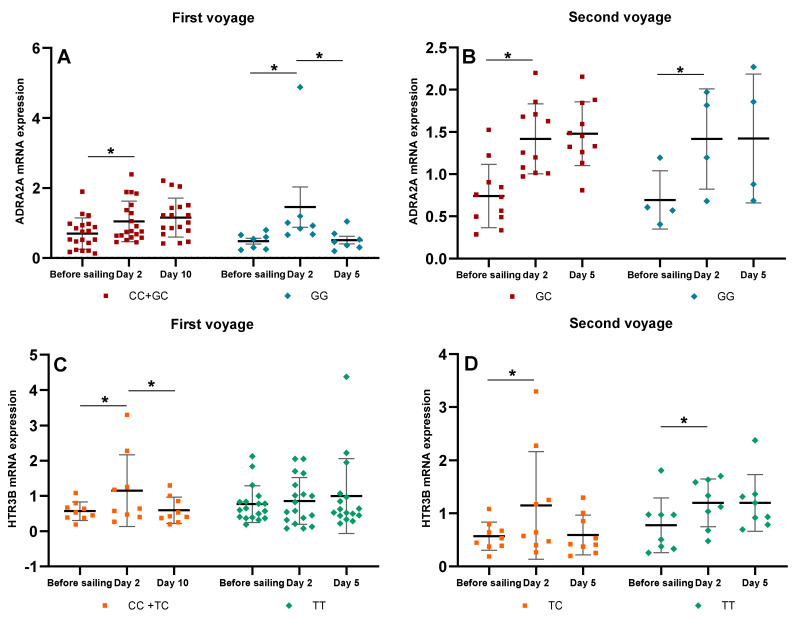
Dynamic changes of ADRA2A and HTR3B mRNA levels in different genotypes on the two voyages. (**A**) ADRA2A mRNA changes of rs1800544 CC+GC and GG genotypes on the first voyage; (**B**) ADRA2A mRNA changes of rs1800544 GC and GG genotypes on the second voyage; (**C**) HTR3B mRNA changes of rs3758987 CC+TC and TT genotypes on the first voyage; (**D**) HTR3B mRNA changes of rs3758987 TC and TT genotypes on the second voyage. * *p <* 0.05; error bar: Standard Deviation (SD).

We then investigated the dynamic changes of ADRA2A and HTR3B mRNA expressions of the subjects with different SNP genotypes. The first voyage lasted for 12 days and the sea state during the whole voyage was kept within degree 2~3, so the subjects were exposed to a lasting and stable low-frequency motion stimulus the entire time. On the tenth day of the voyage most of the subjects’ seasickness symptoms were reduced. The ADRA2A mRNA expression in the rs1800544 GG carriers had a sharp increase on day two (*p =* 0.005) and then decreased significantly on day ten (*p =* 0.006). The CC+GC carriers also had a significant increase of ADRA2A mRNA on day two (*p =* 0.000), but had no significant change on day ten (Figure 4A). The HTR3B mRNA expression was significantly increased in the rs3758987 CC+TC carriers on day two (*p =* 0.004), and then significantly decreased on day ten (*p =* 0.002). The HTR3B mRNA expression of the TT carriers had a continuously increasing trend on days two and ten (Figure 4C).

The second voyage lasted for seven days and the sea conditions became rough on the fourth day, so all the subjects were exposed to 5-degree sea state stimuli. It should be mentioned that there were no CC genotype carriers of ADRA2A rs1800544 and HTR3B rs3758987 on this voyage. The ADRA2A mRNA was significantly increased both in the GG and GC carriers of rs1800544 on day two (*p =* 0.005; *p =* 0.000), and had a slightly continual increase trend on day five (Figure 4B). The HTR3B mRNA was also increased significantly both in the TC and TT carriers of rs3758987 on day two (*p =* 0.000; *p =* 0.000). On day five of the voyage, the HTR3B mRNA of the TC carriers had a slight decrease, and the TT carriers had no significant changes (Figure 4D). The mRNA expressions (Mean ± SD) were shown in Table S2.

**Figure 5:**
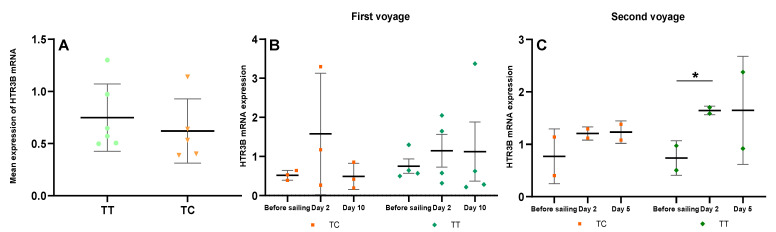
Dynamic changes of HTR3B mRNA level of different rs3758987 genotypes in rs1800544 GG subjects on the two voyages. (**A**) Basic HTR3B mRNA level in different rs3758987 genotype group; (**B**) HTR3B mRNA changes of rs3758987 TC and TT groups on the first voyage; (**C**) HTR3B mRNA changes of rs3758987 TC and TT groups on the second voyage. * *p <* 0.025; error bar: Standard Deviation (SD).

To investigate the dynamic changes of the subjects with two-risk genotypes, we extracted all the rs1800544 GG genotype subjects (11) for further analysis. Among them, on the first voyage, three subjects were carrying the rs3758987 TC genotype and four were carrying the TT genotype. In addition, two subjects were carrying the TC genotype and two were carrying the TT genotype during the second voyage. The basic HTR3B mRNA level in the TT carriers was higher than in the TC carriers (Figure 5A). On the first voyage, the HTR3B mRNA was significantly increased on day two (*p =* 0.034) and decreased on day ten in the rs3758987 TC carriers (*p =* 0.046). while the TT carriers still had a continuously increasing trend on days two and ten (Figure 5B). On the second voyage, the HTR3B mRNA of the TC and TT carriers both increased on day two (*p =* 0.029; *p =* 0.018) and had no significant changes on day five (Figure 5C). The mRNA expressions (Mean ± SD) were shown in Table S3.

## 4. Discussion

The individual differences in motion sickness are not only about the susceptibility, but also the serious extent of the symptoms [43]. Since vomiting is the most typical and intuitive symptom of MS, we take MS-induced vomiting as the main indicator to be observed in the current study. We found that both the ADRA2A rs1800544 and HTR3B rs3758987 polymorphisms had influences on MS-induced vomiting. In this study, two cohorts of subjects participated in the laboratory data collection (n = 315) and the actual-voyage exposure (n = 42). They all declared no history of serious illness or long-term medication, and had similar life backgrounds before they went to college. This effectively reduces the interference of the individual differences induced by complex environmental factors and makes the findings more focused on the differences induced by genetic factors. Since the frequency of the SNPs has obvious differences depending on different ethnicities [44], we chose the Chinese Han population as our subjects to avoid the influence of ethnicity.

We first found that the rs1800544 GG genotype increased the risk for MS, which means, compared to the CC+GC carriers, people carrying the GG genotype were more prone to MS. Then we found that in childhood the rs3758987 CC+TC and rs1800544 GG genotypes increased the risk of MS-induced vomiting. This trend becomes weaker in adulthood and may be because the environmental influence on the reduced MSS is the decisive effect of genes with aging [45].

The ADRA2A gene encodes α2-AR, which is mainly distributed in the presynaptic membrane of the adrenergic neurons in the CNS and plays an important role in signal transduction [46]. They are involved in regulating the release of neurotransmitter molecules from the sympathetic nerves and adrenergic neurons in the central nervous system. A previous study revealed that the GG genotype at the ADRA2A-1296 site was associated with higher MSS in the Chinese population [21]. The NCBI database shows that the G allele at the rs1800544 locus accounts for a higher proportion in the Asian population (G = 0.8, C = 0.2; G = 0.269, C = 0.7307 among Europeans) (NCBI/SNP/rs1800544 [Online]) [47]. Since we found that the rs1800544 G allele carriers are more susceptible to MS (OR = 1.585, 95%CI = 1.136–2.208; Table 2), this site may have the potential to be another parameter of the greater MSS in the Asian population. The SNP of rs1800544 has been reported to be associated with obesity and body fat content and distribution. Whether motion sickness susceptibility has a relation with obesity might be a fun point for future research [48].

The HTR3B gene encodes subunit B of the type 3 receptor for 5-hydroxytryptamine (serotonin), which is a main receptor in mediating vomiting [49]. The SNP of HTR3B rs3758987 was found to serve as a predictor of PONV in the Chinese Han patients undergoing gynecological laparoscopic surgery [36]. Our preliminary research found that rs3758987 and rs1800544 were related to MS-induced vomiting in the Chinese Han population. MS-induced vomiting involves a lot of physiological processes and signal pathways, among them—since MSS seems to be a prerequisite of vomiting—are ADRA2A and HTR3B, which may interact and co-regulate MS-induced vomiting. This hypothesis needs more precise research in the future.

Although the two SNPs of rs1800544 and rs3758987 may not directly affect the amino acid sequence of the encoded protein, they may be in some linkage disequilibrium (LD) with important, but unexplored, functions [50,51]. On the other hand, these SNPs might influence the regulatory processes related to their gene expression and protein function. In this study, we detected their gene expression changes during the two voyages in the different SNP genotypes and found some interesting results.

In the analysis of the ADRA2A and HTR3B gene expression in blood cells, we found that the basic level of ADRA2A mRNA was significantly lower in the rs1800544 GG carriers. The rs1800544 is in the promoter region of the ADRA2A gene, and this may lead to differences in its gene expression [25]. In this study, the ADRA2A mRNA significantly increased within 24 h of the voyages, indicating the activation of α2-AR and leading to a neuroendocrine response [52]. Similarly, the basic level of the HTR3B mRNA was significantly lower in the rs3758987 TC and CC carriers, and then increased within 24 h of the voyages. A previous study reported that when the intestine cells were damaged by motion stimuli, 5-HT in the gut will release and the 5-HT3 receptors are activated, then the impulses are transmitted to the vomiting center via vagal afferents [53]. We, therefore, hypothesized that when subjects were exposed to the actual voyages, their 5-HT3B receptors were activated rapidly and caused vomiting. Furthermore, motion sickness stimulation may trigger systematic inflammation, and the inflammatory processes may induce an up-regulation of white blood cell counts. This might be one of the reasons that the ADRA2A and HTR3B gene expression increased [54]. It has been shown that the immune and nervous systems have bi-directional communication, which coordinates the mobilization of specialized capacities of each system in response to environmental and autologous challenges [55]. The lymphocytes may release peripheral cytokines, which can invoke CNS-controlled autonomic, neuroendocrine, and behavioral responses through several routes. The CNS, in turn, regulates lymphocytes metabolism via hormones, neurotransmitters, and neuropeptides [56]. Therefore, the process of the development of motion sickness may involve an intricate network of connections, which should be paid more attention to in future research.

After a ten-day voyage at calm seas, most of the subjects had alleviated symptoms and they began to habituate to seasickness. The ADRA2A mRNA in the rs1800544 GG carriers decreased to nearly the basic level on day ten. Interestingly, the CC+GC carriers had no such obvious changes, and their mRNA was kept at a higher level during the whole voyage. This may be because the GG carriers are always more susceptible to a motion stimulus, which leads to a bigger change in gene expression. After a long-term voyage, the decreased ADRA2A mRNA, indicating an increase in parasympathetic activity and a restoration of autonomic balance, suggests habituation to seasickness. Moreover, the mRNA increase range in the GG carriers was higher than in the CC+GC carriers, which may be one reason why the GG carriers had more severe symptoms within 24 h of sailing. The HTR3B mRNA in the rs3758987 CC+TC carriers also decreased to the basic level on day ten. While the mRNA of the TT carriers was kept at a stable and higher level in the whole voyage, even on day ten, there was a slight increase trend. This may be a compensatory manifestation of the 5-HT3B receptor, which is to suggest that to maintain homeostasis and avoid excessive excitation of vestibular nucleus neurons, the expression and function of the receptors will be enhanced. Therefore, the TT genotypes may have a stronger regulating ability for gene expression and prevent people from vomiting.

During the second voyage, the increased range difference of the ADRA2A and HTR3B mRNAs on day two was not as significant as the first part between different genotypes. This might be due to the absence of the CC genotype in both SNPs. Although the subjects were exposed to rough seas on day four, their mRNAs had no significant changes compared to day two, and were maintained at a relatively high level during the voyage. Given that the sailing time of this voyage was not as long as the first voyage, and the sea state became rough during the sailing, the subjects didn’t habituate to the seasickness. The high activation of these two receptors might be the reason for the severe seasickness of the subjects.

To explore the synergistic effect of these two SNPs in MS-induced vomiting, we extracted all eleven of the rs1800544 GG subjects for analysis, and got five of the rs3758987 TC and six of the TT carriers. The dynamic changes of the HTR3B mRNA had similar trends to the above findings. Among them, there were four TC carriers (80%), and only one TT carrier (16.7%) who vomited within 24 h of the voyage. This phenomenon strongly implies that people with rs1800544 GG and rs3758987 TC are highly susceptible to MS-induced vomiting. Therefore, the GG+TC haplotype might be a relatively effective predictor for motion sickness. Of course, due to our small sample size, this result still has certain limitations, so it is very worthwhile to continue to expand the sample size for further analysis in future studies.

## 5. Conclusions

In conclusion, our results revealed that the ADRA2A rs1800544 GG genotype is an independent risk factor for MSS. In combination with the incidence of vomiting in real sailing situations, the HTR3B rs3758987 CC+TC and ADRA2A rs1800544 GG genotypes may be involved in the MS-induced vomiting and increase its risk. The high ADRA2A and HTR3B mRNA levels functioned to protect people from seasickness, and their dynamic regulation played an important role in the occurrence and development of seasickness. These results provide novel insight into the genetic underpinnings of MS.

## Figures and Tables

**Table 1:** General characteristics of the subjects in the first part: demographics and percentages of the variables (N = 315).

Variable		All Subjects (315)		Age Group, Year				χ^2^	*p*
				<18		≥18			
		N	%	N	%	N	%		
Age	18.13 ± 0.65	315		38	12.1	277	87.9		
Gender	Men	250	79.4	27	10.8	223	89.2	1.823	0.200
	Women	65	20.6	11	16.9	54	83.1		
MSSQ score	<22.68 (non-susceptibility)	160	50.8	17	10.6	143	89.4	0.634	0.490
	≥22.68 (susceptibility)	155	49.2	21	13.6	134	86.4		

**Table 2:** The ADRA2A rs1800544 and HTR3B rs3758987 gene polymorphisms and risk of motion sickness susceptibility.

SNP	Model		Susceptibility 155 (%)	Non-Susceptibility 160 (%)	χ^2^	*p*	OR (95%CI)
rs1800544		GG	81 (52.3)	58 (36.2)	8.237	0.016	
(G/C)		GC	57 (36.8)	77 (48.1)	6.759	0.009	0.530 (0.328–0.857)
		CC	17 (10.9)	25 (15.7)	4.115	0.043	0.487 (0.241–0.983)
	Recessive	GC+GG	138 (89.1)	135 (84.3)			
		CC	17 (10.9)	25 (15.7)	1.478	0.224	0.665 (0.344–1.287)
	Dominant	GG	81 (52.3)	58 (36.2)			
		CC+GC	74 (47.7)	102 (63.8)	8.183	0.004	0.519 (0.331–0.815)
	Allele	C	91 (29.4)	127 (39.7)			
		G	219 (70.6)	193 (60.3)	7.429	0.006	1.585 (1.136–2.208)
rs3758987		TT	102 (65.8)	95 (59.4)	2.107	0.349	
(T/C)		TC	49 (31.6)	57 (35.6)	0.849	0.357	0.801 (0.499–1.285)
		CC	4 (2.6)	8 (5.0)	1.539	0.215	0.466 (0.136–1.597)
	Recessive	TC+TT	151 (97.4)	152 (95.0)			
		CC	4 (2.6)	8 (5.0)	1.258	0.262	0.503 (0.148–1.707)
	Dominant	TT	102 (65.8)	95 (59.4)			
		CC+TC	53 (34.2)	65 (40.6)	1.390	0.238	0.759 (0.480–1.200)
	Allele	T	253 (81.6)	247 (77.2)			
		C	57 (18.4)	73 (22.8)	1.883	0.170	0.762 (0.517–1.124)

**Table 3:** The HTR3B rs3758987 and ADRA2A rs1800544 gene polymorphisms and risk of motion sickness induced-vomiting in childhood.

SNP	Model		Vomited 181 (%)	Non-Vomited 134 (%)	χ^2^	*p*	OR (95%CI)
rs3758987		TT	104 (57.5)	93 (69.4)	7.730	0.021	
(T/C)		TC	72 (39.8)	34 (25.4)	6.482	0.011	1.894 (1.155–3.105)
		CC	5 (2.8)	7 (5.2)	0.561	0.454	0.639 (0.196–2.081)
	Recessive	TC+TT	176 (97.2)	127 (94.8)			
		CC	5 (2.8)	7 (5.2)	1.273	0.259	0.515 (0.160–1.661)
	Dominant	TT	104 (57.5)	93 (69.4)			
		CC+TC	77 (42.5)	41 (30.6)	4.689	0.030	1.679 (1.049–2.690)
	Allele	T	280 (77.4)	220 (82.1)			
		C	82 (22.6)	48 (17.9)	2.114	0.146	1.342 (0.902–1.998)
rs1800544		GG	89 (49.2)	50 (37.3)	5.508	0.064	
(G/C)		GC	73 (40.3)	61 (45.5)	2.580	0.108	0.672 (0.414–1.092)
		CC	19 (10.5)	23 (17.2)	4.732	0.030	0.464 (0.231–0.934)
	Recessive	GC+GG	162 (89.5)	111 (82.8)			
		CC	19 (10.5)	23 (17.2)	2.962	0.085	0.566 (0.294–1.088)
	Dominant	GG	89 (49.2)	50 (37.3)			
		CC+GC	92 (50.8)	84 (62.7)	4.391	0.036	0.615 (0.390–0.970)
	Allele	C	111 (30.7)	107 (39.9)			
		G	251 (69.3)	161 (60.1)	5.838	0.016	1.504 (1.079–2.092)

## Data Availability

Data available on request due to restrictions e.g., privacy or ethical.

## Supplementary Materials

The following are available online at https://www.mdpi.com/article/10.3390/ijerph182413163/s1, Figure S1: The genotyping results of PCR-RFLP. Table S1: mRNA expression of ADRA2A and HTR3B in all subjects. Table S2: mRNA expression of ADRA2A and HTR3B in all subjects with different genotypes in two voyages. Table S3: mRNA expression of HTR3B mRNA level of different rs3758987 genotypes in rs1800544 GG subjects in two voyages.
