# Supplementary material for: The Predictive Role of ADRA2A rs1800544 and HTR3B rs3758987 Polymorphisms in Motion Sickness Susceptibility
Source: Int J Environ Res Public Health. 2021 Dec 14;18(24):13163. doi: 10.3390/ijerph182413163 (PMC8701240; doi:10.3390/ijerph182413163)
Supplement: Supplementary file 1 [file ijerph-18-13163-s001.zip › ijerph-1448183-supplementary.pdf]

Supplemental Information

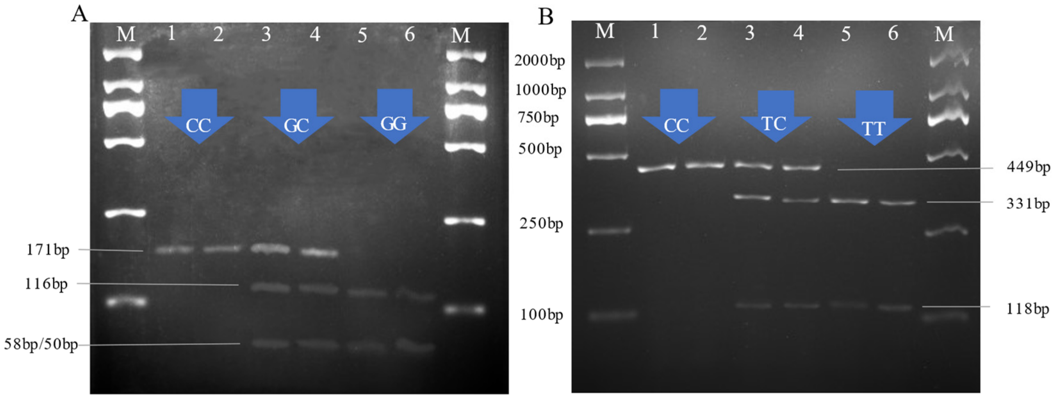

Figure S1. The genotyping results of PCR-RFLP. (**A**) Show the products of ADRA2A PCR-RFLP; (**B**) show the products of HTR3B.

Table S1. mRNA expression of ADRA2A and HTR3B in all subjects.

		**Mean ± SD**
ADRA2A	CC	0.885
	GC	0.708 ± 0.423
	GG	0.559 ± 0.277
	Before sailing	0.673 ± 0.388
	24 h after sailing	1.245 ± 0.775
HTR3B	TT	0.771 ± 0.510
	TC	0.593 ± 0.245
	CC	0.491 ± 0.432
	Before sailing	0.696 ± 0.437
	24 h after sailing	1.041 ± 0.677

Table S2. mRNA expression of ADRA2A and HTR3B in all subjects with different genotypes in two voyages.

**ADRA2A**		**Mean ± SD**	
		**GC + GG**	**GG**
First voyage	Before sailing	0.698 ± 0.447	0.481 ± 0.223
	Day 2	1.046 ± 0.579	1.439 ± 1.484
	Day 10	1.154 ± 0.557	0.511 ± 0.285
Second voyage	Before sailing	0.742 ± 0.375	0.695 ± 0.344
	Day 2	1.418 ± 0.414	1.416 ± 0.593
	Day 5	1.479 ± 0.378	1.423 ± 0.762
HTR3B		Mean ± SD	
		TC + CC	TT
First voyage	Before sailing	0.568 ± 0.265	0.769 ± 0.521
	Day 2	1.150 ± 1.017	0.855 ± 0.664
	Day 10	0.593 ± 0.374	0.997 ± 1.063
Second voyage	Before sailing	0.595 ± 0.264	0.775 ± 0.518
	Day 2	1.253 ± 0.212	1.197 ± 0.452
	Day 5	1.109 ± 0.195	1.198 ± 0.534

Table S3. mRNA expression of HTR3B mRNA level of different rs3758987 genotypes in rs1800544 GG subjects in two voyages.

ADRA2A(GG)		HTR3B Mean ± SD	
		TC	TT
		0.620 ± 0.308	0.749 ± 0.322
First voyage	Before sailing	0.520 ± 0.126	0.754 ± 0.369
	Day 2	1.578 ± 1.554	1.148 ± 0.832
	Day10	0.489 ± 0.334	1.375 ± 2.006
Second voyage	Before sailing	0.771 ± 0.521	0.738 ± 0.330
	Day 2	1.205 ± 0.124	1.644 ± 0.083
	Day 5	1.232 ± 0.214	1.747 ± 1.031
